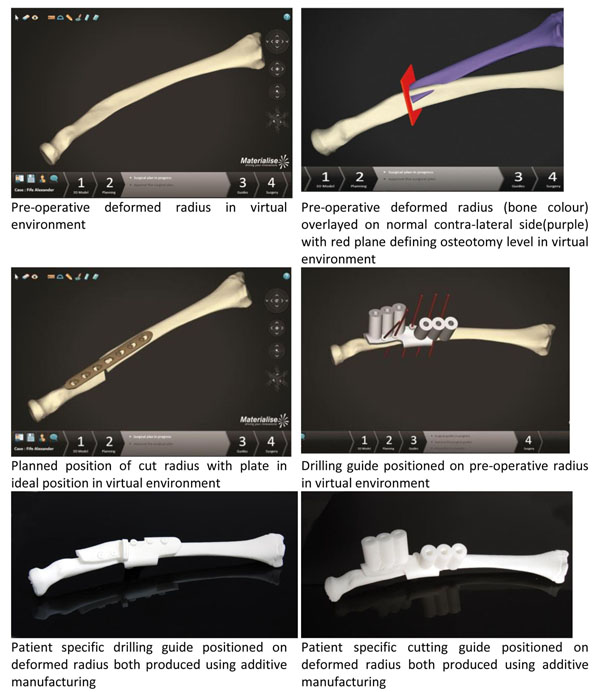# Case studies using 3D technologies for corrective osteotomies : synergy between engineer and surgeon

**DOI:** 10.1186/1753-6561-9-S3-A92

**Published:** 2015-05-19

**Authors:** Els Bryunooghe

**Affiliations:** 1 Materialise, Antwerp, 2930, Belgium

## 

Three-dimensional (3D) visualization, surgical pre-planning, and 3D printing or additive manufacturing of patient specific instruments and implants are fast gaining attention in the medical world. Already widely used in cranio-maxillofacial, dental, and total knee replacement surgery, 3D technologies are now starting to create a trend in the upper limb region too.

A crucial step in establishing successful synergy between 3D technology and the orthopaedic world is to thoroughly get to know one another. Technology companies should constantly be aware of the changing needs of the medical field and be ready to evolve where and when needed. The medical field, in turn, should be aware of the new possibilities this technology offers but also mindful of its limitations. This is true in general and maybe even more so for corrective osteotomies in the upper limb, where current techniques mostly rely on two-dimensional(2D) X-rays and mass-standardized implant designs to solve often complex and extremely individualized malformations.

In day to day practice, this synergy starts with an orthopaedic surgeon virtually planning his/her surgery together with the (clinical) engineer. Using Materialise solutions (Materialise N.V, Leuven, Belgium), nearly 500 upper-and lower limb cases have been completed with this personalized approach globally. It requires the surgeon and the engineer to, ‘sit together’, as it were, during online web sessions to discuss the case and to find the preferred solution for each individual patient.

Applications for this 3D technology range from mal-united distal and diaphyseal extra-articular fractures to intra-articular fractures with more than 2 distal fragments, rare forms of radio-ulnar synostosis, cubitus varus/valgus, multiple hereditary exostosis, and Madelung deformities. Preparing for corrective osteotomies with the use of digital pre-planning is, in most cases, complemented with 3D printed guides. With engineering experience in approximately 180 of such cases, a few case studies are selected to highlight virtual planning, its possibilities, and its consequences.

A common approach in the virtual pre-planning of upper limb corrective osteotomies is having a 3D visualization of both limbs based on a CT scan. The 3D visualization is then manipulated to reveal the comparison between the healthy – if any – and the surgical side, illustrating very clearly the deformity and the correction needed. Decisions are made with respect to the amount of lengthening or shortening required, whether the intra-articular component needs to be addressed, where to perform the osteotomy, which plate to use, and many more. This is all done with a focus on the original pathology that must be addressed. Often, this step is an eye-opening diagnostic tool as many 3D deformities are not captured on X-ray.

Virtual 3D planning provides an opportunity to digitally “test out” several different approaches for the surgery, resulting in a final, preferred solution that can then be brought to the OR. However, to reach this optimized surgical plan, there is a high need for synergy between the clinical knowledge and experience of the surgeon and the technical knowledge and experience of the engineer involved in the planning. When this synergy is established, patients get treatments with a single, full-contact osteotomy, or with optimized wedges for grafting. In some cases, patients are finally treated for a pathology that was previously deemed impossible to treat with conventional, standard techniques.

The potential applications of this technology are boundless, but the limitations must be carefully considered as well. For example, simulation of the behavior of the soft tissue after correction still remains uncertain. Although the goal of the surgical plan is to achieve a bony anatomical restoration, there is no guarantee that the soft tissues will allow this bony restoration to regain full pro-supination and/or flexion-extension. Additionally, in cases where a healthy contralateral side is unavailable or bilateral congenital deformities are involved, the surgical plan is even more dependent on the surgeon’s experience to correct the malunion in the most suitable way.

Provided each other’s strengths and limitations are taken into consideration, synergy between an engineer and a surgeon can become the foundation for revolutionizing the way orthopaedic surgery is done. It is clear the future holds more exciting new opportunities.

**Figure 1:**